# Fabrication of micro-ridge long-period gratings inscribed on polarization-maintaining fibers

**DOI:** 10.1186/1556-276X-9-39

**Published:** 2014-01-20

**Authors:** Oh-Jang Kwon, Myungjun Shin, Young-Guen Han

**Affiliations:** 1 Department of Physics, Hanyang University, 17 Haengdang-dong, Seongdong-gu, Seoul 133-791, South Korea

**Keywords:** Long-period fiber gratings, Strain sensor, Polarization-maintaining fiber

## Abstract

We experimentally investigated a simple and new technique for the fabrication of micro-ridge long-period gratings (MRLPGs) based on polarization-maintaining fibers (PMFs). The cladding region of the PMFs was etched periodically using a wet etching technique resulting in the periodic formation of micro-ridges on the surface of the PMF. The PMF-based MRLPGs has two resonant peaks because of the birefringence of the PMF. The extinction ratios of two resonant peaks of the PMF-based MRLPGs were effectively improved by increasing the applied strain because of the photoelastic effect.

## Background

Long-period fiber gratings (LPGs) have attracted much attention in optical communication systems and optical sensors because of their many advantages, such as low cost, ease of fabrication, and electromagnetic immunity [1-3]. Since the cladding modes coupled from the guided core mode in the LPGs are directly interfaced with external environments, the LPGs have high sensitivity to ambient perturbation change such as temperature, strain, and ambient index [1-3]. In general, UV excimer lasers and frequency-doubled argon lasers are conventionally exploited to fabricate the LPGs based on the variation of the photoinduced refractive index [1-3]. For specialty fibers without photosensitivity, such as photonic crystal fibers, however, it is not easy to induce the refractive index change with UV excimer lasers and frequency-doubled argon lasers. Recently, the LPGs inscribed on a dispersion-shifted fiber (DSF) by etching its silica-based cladding with the hydrofluoric acid (HF) solution after taking the metal coating process was proposed [4]. However, it is difficult to symmetrically deposit the metal layer on the silica-based cylindrical cladding of the DSF. In this paper, we propose a new fabrication technique of the micro-ridge long-period gratings (MRLPGs) using both wet etching and double polymer coating methods. In addition, a polarization-maintaining fiber (PMF), for the first time to our knowledge, is implemented to make the MRLPGs. The birefringence of the PMF generates two resonant peaks in the transmission spectrum of the PMF-based MRLPGs. The applied strain changes the extinction ratio of two resonant peaks but not their wavelengths because of the photoelastic effect. It means that the proposed PMF-based MRLPGs have the great potential for the application to strain sensors.

## Methods

Mode coupling in the MRLPGs is based on the photoelastic effect. After the formation of the periodic micro-ridges in the cladding of the optical fiber, the different cross-sections between the etched and the unetched claddings can essentially induce the periodic index modulation based on the photoelastic effect when strain is applied to the optical fiber [4]. Consequently, the resonant peak in the transmission spectrum resulting from the mode coupling between the core and the cladding modes in the MRLPGs can be created by applying strain. The transmission of the MRLPGs (*T*) can be written as [4]

(1) $$T≅\cos^{2}\left[p_{e}\left(\frac{{r_{u}}^{2}}{{r_{e}}^{2}}-1\right)\mathrm{\epsilon l}\right]$$

where *p*_*e*_ is a photoelastic coefficient, *r*_e_ and *r*_u_ are the radii of the etched and the unetched regions, respectively, *ϵ* is the applied strain, and *l* is a grating length. Since the periodic micro-ridges are structurally formed in the cladding region, the averaged cladding mode index should be considered and the structural index change in the core region is negligible [4]. When the MRLPGs are inscribed on the PMF, the transmission spectrum strongly depends on the birefringence of the PMF when strain is applied to the PMF-based MRLPGs. By taking into account the birefringence of the PMF considering (Δ*n*_PMF_), the resonant wavelength of the PMF-based MRLPG can be written as

(2) $$\lambda_{\mathrm{res}}=\frac{\Lambda \left(\Delta n_{\mathrm{PMF}}-{\bar{n}}_{\mathrm{cl}}\right)}{1+\left({\bar{\kappa }}_{\mathrm{cl}}-\kappa_{\mathrm{co}}\right)\Lambda /2\pi }$$

where ${\bar{n}}_{\mathrm{cl}}$ is the averaged effective index of the cladding mode, *Λ* is a grating period, and ${\bar{\kappa }}_{\mathrm{cl}}$ and *κ*_co_ are the averaged self-coupling coefficients of the cladding and core modes, respectively. From Equation 2, it is evident that the resonant wavelengths should be determined by the birefringence of the PMF [5].

**Figure 1:**
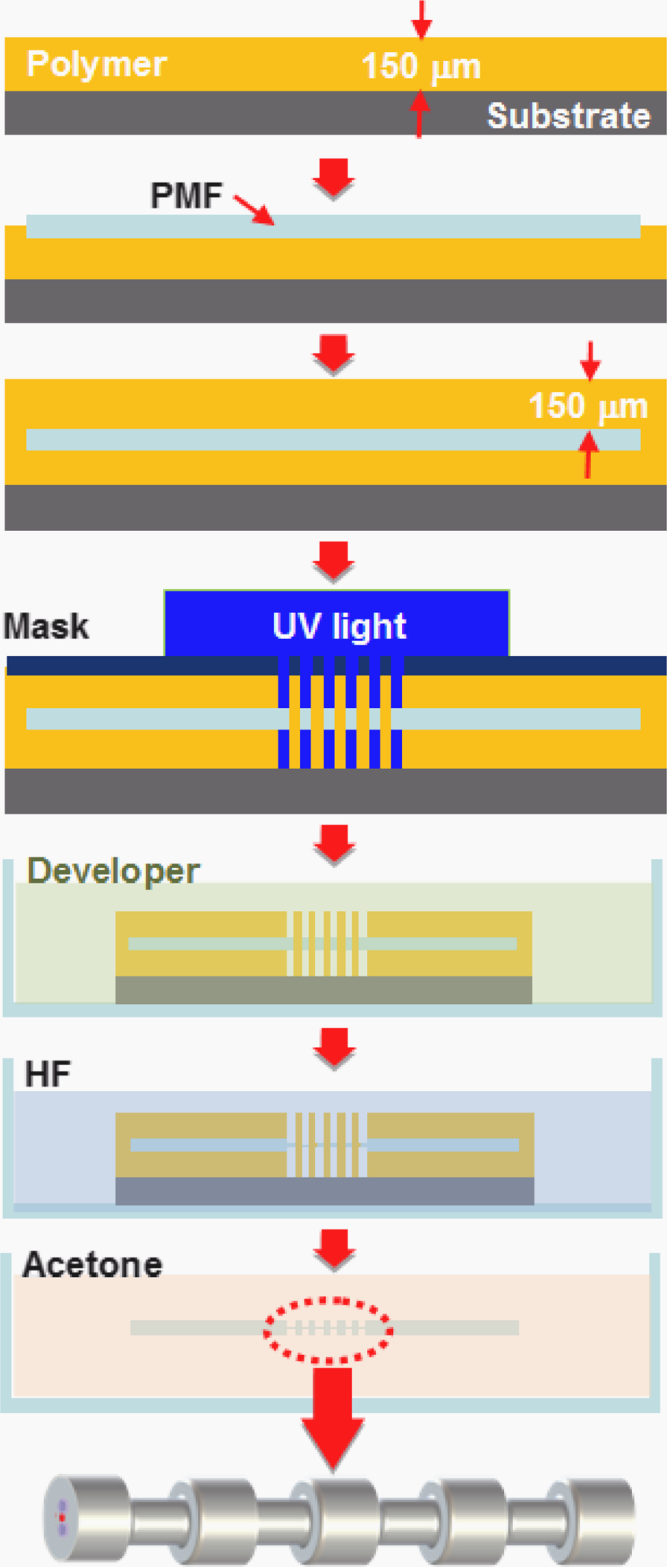
Fabrication process of the PMF-based MRLPG using the double polymer-coating and wet etching techniques.

Figure 1 exhibits the fabrication procedure of the PMF-based MRLPG using the double polymer-coating and wet etching methods. The polymer (PCA-3000 PM) with a thickness of 150 μm was firstly coated on the substrate using a spin coater. After aligning the PMFs (SM.15-P-8/125-UV/UV-400, Fujikura, Chiba, Japan) on the surface of the substrate with the polymer coating, we completely covered the PMFs with the same polymer using a spin coater again. The solvent within the polymer was vaporized using a hot plate. The PMF with doubly coated polymer layers was periodically exposed to UV light through an amplitude mask with a length of 20 mm and a grating period of 550 μm, respectively. The polymer patterns on the surface of the PMF were periodically remained after eliminating the UV-light-exposed polymer using a developer of P-7G. The periodicity of the polymer patterns that may protect the PMF from being engraved by the HF solution should be determined by that of the amplitude mask. The PMF with the periodic polymer patterns was immersed in the HF solution to etch the silica surface of the PMF resulting in the formation of the periodic micro-ridges on the surface of the PMF. The remained polymer was removed using the acetate solution. Consequently, the LPG with periodic ridge structures on the surface of the cladding of the PMF could be realized.

## Results and discussion

**Figure 2:**
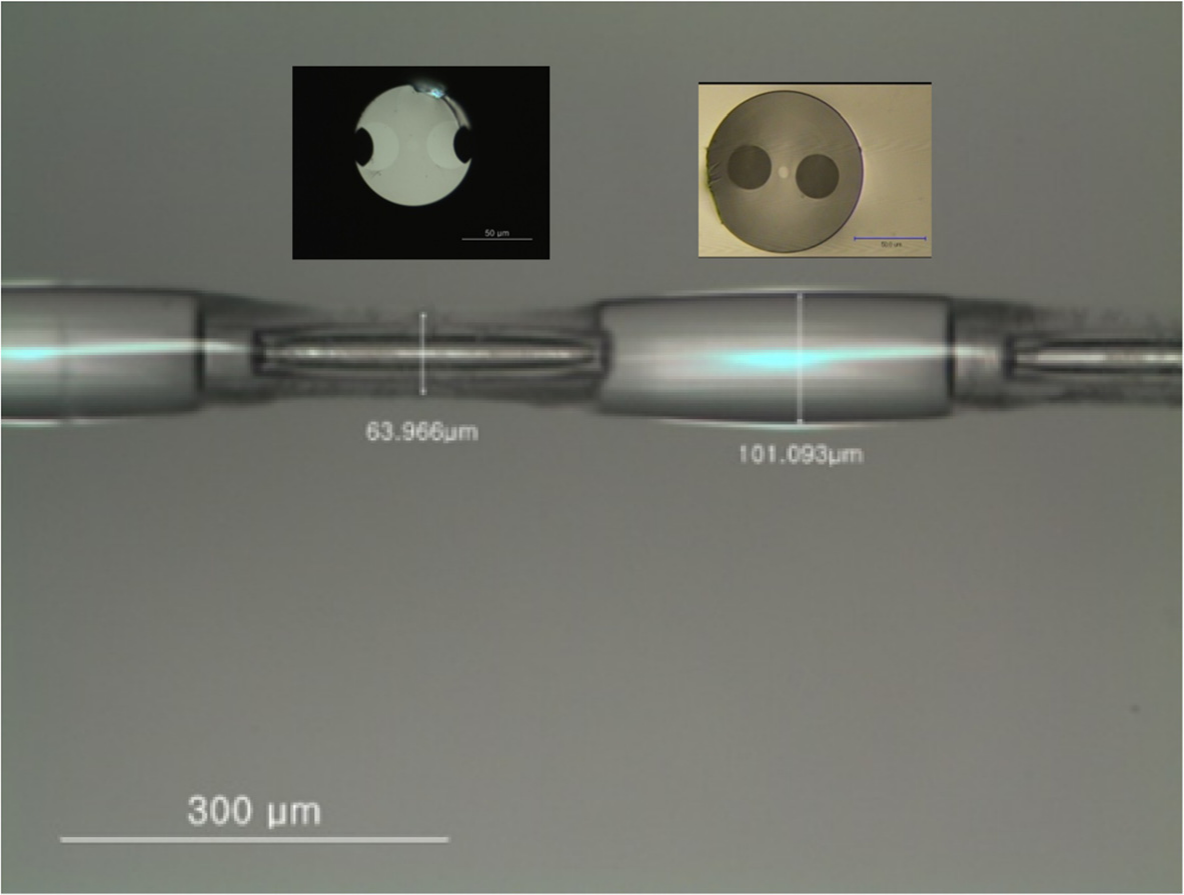
Photograph of the fabricated PMF-based MRLPG measured using an optical microscope.

Figure 2 depicts the photography of the fabricated PMF-based MRLPG measured using an optical microscope. It is clearly obvious that the silica cladding of the PMF was periodically etched by the HF solution and the periodic micro-ridges were developed in the PMF. Since the silica cladding without the polymer coating in the PMF was corroded by the HF solution, its diameter should be reduced. The stress bars inside of the PMF were partially removed in the etched regions because the B_2_O_3_-based stress region was etched higher than that of the silica cladding [6,7]. The diameters of the etched and unetched region were measured to be approximately 64 μm and approximately 101 μm, respectively. The grating period was measured to be approximately 550 μm, which was the same as that of the amplitude mask.

**Figure 3:**
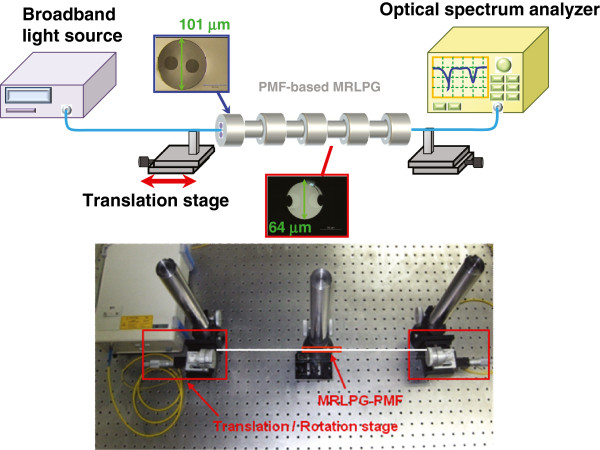
Experimental setup for measurement of the transmission characteristics of the PMF-based MRLPG.

The periodic micro-ridges in the PMF can induce the periodic index modulation based on the photoelastic effect resulting in the mode coupling between the core and the cladding modes when strain is applied. The transmission characteristics of the PMF-based MRLPG were measured using the experimental setup as shown in Figure 3. The measurement setup consists of a broadband light source, linear translation stages, and an optical spectrum analyzer. Both ends of the PMF-MRLPG were clamped by two linear translation stages. A distance between two translation stage was 30 cm. Strain was applied by moving the translation stage outwards.

**Figure 4:**
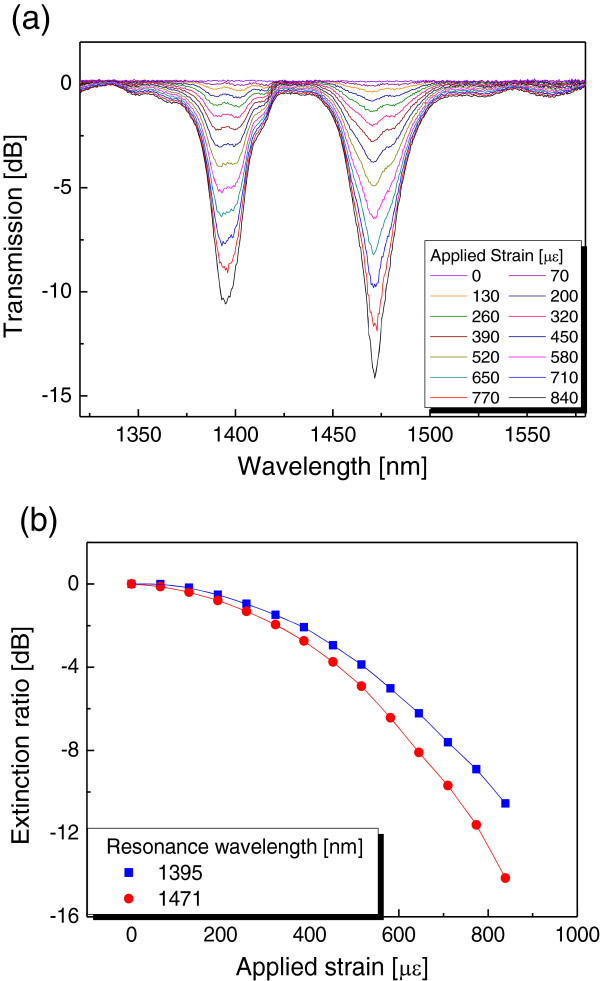
Transmission spectra (a) and variation of extinction ratios of two resonant peaks (b) of PMF-based MRLPG.

Figure 4a shows the transmission spectra of the fabricated PMF-based MRLPG with variations in strain. The birefringence of the PMF generated two resonant peaks in the transmission spectrum of the PMF-based MRLPG when strain was applied. Since the mode coupling between core and cladding modes based on the photoelastic effect is enhanced by increasing strain, the extinction ratio of the PMF-based MRLPG is obviously raised by strain. Two resonant wavelengths of the PMF-based MRLPG corresponding to two orthogonal polarization states were measured to be 1,395 and 1,471 nm. In Figure 4b, the variations of extinction ratios of two resonant peaks at wavelengths of 1,395 and 1,471 nm were measured to be −10.16 and −14.13 dB, respectively, when the applied strain was 840 μϵ. However, two resonant wavelengths were almost not changed by the applied stain because the photoelastic effect was simultaneously induced in the core and the cladding regions. When strain is applied to the PMF-based MRLPG, the variations of the effective refractive indices in the core and the cladding regions are almost identical, which induces the same amount of two self-coupling strengths in the core and the cladding modes [4]. It means that the proposed PMF-based MRLPGs can provide a simple sensing scheme for measurement of strain by monitoring the transmission power variation with respect to the external strain change.

## Conclusion

We proposed and experimentally demonstrated a fabrication method for the PMF-based MRLPG using the double coating and the wet etching processes, which has the great potential for mass production. The transmission characteristics of the PMF-based MRLPG with variations in strain were measured. Two resonant peaks of the PMF-based MRLPG were observed in the transmission spectrum of the PMF-based MRLPG because of the birefringence of the PMF. The extinction ratios of two resonant peaks of the PMF-based MRLPG were enhanced by increasing the applied strain without variation in their resonant wavelengths because of the photoelastic effect. The variation of the extinction ratios of two resonant peaks at wavelengths of 1,395 and 1,471 nm were measured to be −10.16 and −14.13 dB, respectively, when the applied strain was 840 μϵ. We believe that the experimental results are very useful for applications to fiber optic sensors, optical switch filters, etc.

## Competing interests

The authors declare that they have no competing interests.

## Authors’ contributions

O-JK and MS participated in the experimental fabrication. Y-GH wrote and corrected the manuscript and conceived and supervised the study. All authors read and approved the final manuscript.
